# Reviews and Book Notices

**Published:** 1877-09

**Authors:** 


### Reviews and Book Notices.

Guy's Hospital Reports; Third Series, Vol. XXII. Published by *J. & A. Churchill*, New Burlington St., London, 1877.

The first article in this Report is by the veteran Edward Cock, on the laws governing the induration of primary syphilitic sores. From extensive personal observation, he asserts that the greatest induration occurs when the sores are situated on regions possessing the greatest amout of loose areolar tissue. Hence upon the prepuce induration proceeds to the greatest extent; on the body of the penis it is much less, and on the surface of the glans least of all.

From comparison of a vast number of recorded cases of syphilis, Mr. Cock concludes that the location of the chancre has a very important influence on the obstinacy and severity of the secondary symptoms, and in the following order:

1. Chancres on the outer edge of the prepuce have very slight secondary symptoms, but produce somewhat severer ones if they are near its base.

2. Sores on the body of the penis are followed by more severe secondary consequences, and still more so as the sore is nearer the pubis.

3. Chancres on the glans produce more secondary symptoms than those on the prepuce, or on the body of the penis, and, if situated in the groove behind the corona, the secondary results are the worst of all.

4. These inductions seem to imply that secondary syphilis is severe in proportion to the closeness of the anatomical relations of the chancre with the general system.

If these points shall be established they will prove germ thoughts, from which will spring a harvest of practical deductions.

The second article is by Samuel Wilks, M. D., on cerebritis, hysteria, and bulbar paralysis.

Dr. George H. Savage follows in an essay on "Some Relations of mental disease to Inheritance."

Dr. Pye-Smith gives us thirty-six pages on "Zanthelasma," and Mr. C. Higgens eighteen pages on "Preventable Blindness."

After these comes an elaborate article on the classification and distribution of diseases, and one on skin diseases, illustrated with plates. Dr. Goodhart gives an article of over eighty pages on the treatment, etc., of empyema. He tabulates seventy-seven cases. The favorite treatment was subaqueous drainage, by means of a drainage tube opening in a vessel of water. Thirty-one of the cases were treated in this way, with thirteen deaths, or about forty per cent. mortality. It appears that the Chicago plan of drainage and daily antiseptic injections, which has been applied here in about ten cases without a single failure, has scarcely occurred to the surgeons of Guy's Hospital, and they still persist in a method which terminates in the death of nearly half of the patients. They report only one case treated by injections, and say nothing as to what the injected materials were. Dr. Andrews, of Chicago, commenced the antiseptic pleural injections and published his cases four or five years ago. Dr. Fox, of Riverside, Ill., and Drs. Davis and Vittum, of Barraboo, Wis., have practiced it, and later it has been adopted in most of the American cities. It is scarcely creditable, for the surgeons of the greatest city in the world, to omit the use of a method which is everywhere else so much more successful than their own plan.

The remainder of the volume contains articles on Bright's, Addison's and Hodgkin's diseases, on osteotomy of the neck of the femur; on disease of the cæcum, on punctured fracture of the skull, on myeloid and several other diseases of the bones; on diphtheria and croup; on the use of sedatives, on the nervous system in diabetes; on poisoning by phosphorus; and on the treatment of varicose veins by excision.

The volume closes with a number of short communications, and with a statistical analysis of the patients treated during the year. The total number treated in the wards was about 5,700, and the out-patients prescribed for were about 14,000.

The work is illustrated with quite a number of lithographs, and is a valuable contribution to the professional literature.

A COURSE OF PRACTICAL HISTOLOGY: BEING AN INTRODUCTION TO THE USE OF THE MICROSCOPE. By *Edward Albert Schaefer*, Assistant Professor of Physiology in University College, London. With illustrations on wood. Philadelphia: *Henry C. Lea*, 1877.

The author says in his preface that "THE PURPOSE of this work is to afford to those engaged in the practical study of Histology, plain and intelligible directions for the suitable preparation of the animal tissues; with the object, either of immediate study, or of their preservation as specimens for future reference." A sensible aim, surely.

The book begins with an introduction, describing, briefly but clearly, the various forms of microscopes of use to those engaged in Histological work. He omits all discussion of the optical principles involved in the construction of the instrument, and goes at once to a description of its parts and their uses. He makes no invidious distinctions, and mentions no names of makers, but the instrument which he figures is of the continental pattern, probably the small stand of Hartnack. He also figures and describes some of the more useful instruments in the preparing and mounting of objects, as forceps, and scissors of various forms, needles set in handles, etc. The introduction ends with a few brief, general directions for work.

In Chapter I. he begins with a study of the blood, giving directions for the examination of a drop of blood as it is drawn from the finger, and describing the different structures to be seen, and how to observe them. He then passes to the consideration of the action of different re-agents upon blood, beginning with heat, and illustrates, among other and older ones, a new and very ingenious apparatus for keeping blood or any other substance, at an even high temperature under the microscope for an indefinite time. He then passes on to the action of water, acetic acid, tannic acid, alkalies and chloroform upon the blood.

In submitting the blood to the action of the latter substance, he uses the moist chamber of Stricker; this chamber is made of a ring of putty put upon the slide. In using the chamber, a drop of chloroform is placed within the ring, and a thin glass with a drop of blood adhering to its under side, is placed over the ring, so that the drop of blood and of chloroform are within the same chamber. He also describes a plan for submitting blood to the vapor of chloroform, or other substances; he attaches a bit of glass tubing to the slide with a little sealing-wax, the end of which extends within the moist chamber; to the outer end of this glass tube a rubber tube is connected, which leads from the source of the supply of the vapor.

Leaving the consideration of human blood, he passes to the examination of frog's blood, and the "feeding of the white corpuscles" by rubbing up Indian ink with salt solution and mixing it with blood. The action of boracic acid and of carbonic acid gas upon frog's blood are next described, and a very ingenious apparatus for investigating the action of electricity upon the blood. The chapter closes with some account of the chemical constitution of the corpuscles.

This chapter is one of the most complete and satisfactory of any with which we are acquainted upon the subject.

Chapter II. goes in the same thorough manner into the study of epithelium. First, he considers the scales of epithelium found in saliva, the deep layers of the epithelium of the mouth, the horny layer of the epidermis, the columnar epithelium of the intestinal canal. He next passes on to the study of ciliated epithelium, recommending for this purpose the gills of the sea water mussel, and gives a number of interesting observations as to the action of various re-agents upon the motions of the cilia, such as heat, weak alkalies, carbonic acid and chloroform; he then considers the form and structure of the cells and the manner of preserving them, as permanent objects.

These chapters may serve as samples of the book; we will merely indicate the contents of the remaining chapters.

Chapter III. is taken up with a consideration of connective tissues, chapter IV. with cartilage, chapter V. with bone, chapter VI. with muscular tissue, chapter VII. with nervous tissue, including the nervous endings.

In this connection we would like to have seen a reference to the method of preparation so long used and defended by Dr. Beale. His methods certainly deserve more consideration than they have received in certain quarters. Many of his conclusions have been corroborated by other observers—indeed nearly all of his easier observations have been so confirmed, and the results of investigations are approaching nearer and nearer to his conclusions, and we are sure that they will in the main, be all confirmed in time. Dr. Beale's peculiar method of preparation is difficult, requiring skill and patient attention to many details, as well as the use of the very highest powers of the microscope. This may, perhaps, explain in some degree, the want of success of other observers, in reaching his results, and the neglect with which his views have been often treated.

Chapter VIII. describes the method of studying blood vessels; cutting sections of tissues; the examination of the circulation of the blood in the web, and mesentery of the frog, in the mesentery of small mammals, and in the lung and tongue of the toad. He then enters upon the consideration of methods of injecting, and the apparatus to be used. Chapter IX. is concerned with the lymphatics and serous membranes; chapter X. with the skin, hair and nails; chapter XI. takes up the heart; chapter XII. the lungs; chapter XIII. the mouth and pharynx; chapter XIV. the esophagus and the stomach; chapter XV. the small and large intestines; chapter XVI. the liver; chapter XVII. the spleen and urinary organs; chapter XVIII. the generative organs; chapter XIX. the central nervous system, the brain and spinal cord; chapter XX. the eye; chapter XXI. the ear and organs of taste. In conclusion there is an appendix with directions for measuring objects under the microscope, drawing objects, counting blood corpuscles, etc.

As a whole, the book is an admirable one. The descriptions are brief, but they are clear and detailed. The author has learned that rare art of stopping when he has said his say. The book has also the still more uncommon merit of bearing everywhere the impress of the author's own thought.

There is no such thing in the book as a condensation of a chapter, or section, or paragraph, from any one else. Even when describing some of the commonest processes, he shows such a practical familiarity with the details as to give his description the flavor of originality.

There is no useless lumber about the book; antiquated chapters, obsolete cuts, and pet theories are relentlessly pruned away, to the great saving of the reader's time and patience, and the great improvement of the whole work.

The illustrations are few in number, only about forty, besides the one which faces the title page, but they always serve a useful purpose. None are introduced for the sake of looking pretty.

In conclusion, we can confidently recommend the work as the most useful manual for the practical histologist with which we are acquainted. L. C.

How to Use the Ophthalmoscope. Being elementary instructions in Ophthalmoscopy. Arranged for Students. By *Edgar A. Browne.* Philadelphia: *Henry C. Lea.* 1877.

About twenty-five years ago, the ophthalmoscope was presented to the medical profession; it has, during its short career, accomplished wonderful things—results equalled by but few, and surpassed by no other instruments in a physician's hands. And yet, instead of being a pocket companion of most physicians, the ophthalmoscope is rarely found outside of the office of an oculist!

Why is it that this valuable little instrument is so unpopular with the average practitioner? In the first place, they think everything relating to the eye does not concern the ordinary physician, and consequently, do not care to know anything about it. And then, they have the idea that there is a certain mysterious difficulty in the use of the ophthalmoscope, that ordinary mortals cannot master.

Now, we contend that the average student of medicine is capable of learning the art of ophthalmoscopy, if he begins at the beginning and proceeds slowly and systematically. As the child has to learn the A, B, and C before it can read, so the student must acquaint himself with the elementary facts in ophthalmoscopy, before he can use the instrument for any particular diagnostic purpose. He must know the fundamental laws of optics; he must learn how to look into the eye, and what to see in the normal eye. If these elements of the physical diagnosis of the eye are presented in plain and intelligent language, even the rawest student will be enabled to comprehend them. But the fault of many teachers is their desire to teach very much in the shortest possible time; they hurry, within the same lecture, from physical diagnosis to the symptomatology of diseases; from the appearance of a healthy eye to morbid anatomy. The student thus is suddenly bewildered by a multitude of new facts. This feeling will not overcome one who has read Browne's little book of 120 small octavo pages. It is very elementary; it is the very A, B, C of ophthalmoscopy, and for this reason we would recommend it to medical students. Indeed, we wish it might make many friends, because we expect it will do much toward popularizing the use of the ophthalmoscope.

The book, however, has a few deficiencies which should not pass unnoticed.

On page 58, Figs. 24 and 25 are designed to show the effect of inversion of the ophthalmoscopic image. The explanatory note to Fig. 24 reads as follows: "Fig. 24 is a map of the left eye in the erect image, showing the position of a disk, with the largest vein curving upward, and a diseased patch in the upper and inner quadrant of the field." But this diseased patch is situated in the sketch to the *right* side of the disk which always corresponds to the *outer* half of the *left* retina, and consequently the above explanation should read: "*a diseased patch in the upper and outer quadrant.*"

Speaking of the examination of the erect image the author says (p. 59): "Ascertain, in the first place, that your own refraction is normal; if not, a suitable correcting lens must be placed in the clip behind the mirror." He should have added right there, that it is equally important to ascertain the refraction of the examined eye and to correct any refractive anomaly, especially myopia, by a suitable glass. With such directions as given on pages 54 to 61, a student will never succeed in seeing the erect image of the fundus of a near-sighted eye.

A serious blunder, we think, is committed by leaving out the parallactic displacement, among the signs by which we are able to ascertain alterations in the surface-level in the fundus. It is of far greater diagnostic value than any of the signs Browne has mentioned.

We notice a single typographical error: "Stannings papille" instead of "Stauungspapille."

COMPENDIUM OF HISTOLOGY. Twenty-four lectures by *Heinrich Frey*, Professor. Translated from the German by permission of the author, by *George R. Cutter*, M. D., Assistant Surgeon New York Eye and Ear Infirmary; Ophthalmic and Aural Surgeon, New York Dispensary, etc., etc. Illustrated by 208 engravings on wood. New York: *G. P. Putnam's Sons.* 1876.

This book is a weariness to the flesh; a plague to the conscientious reviewer. It is a book one takes up with expectation and delight, reads with a sense of disappointment, and throws aside with a feeling of profound relief at having completed an irksome task. Its style is stilted, bombastic, sophomoric. Take for example the first sentence of the First Lecture: "A deep abyss separates the inorganic from the organic, the inanimate from the animate. The rock-crystal on the one side—vegetable and animal on the other; how infinitely different the image." We almost experience a feeling of relief when we find that no convulsion of nature follows the promulgation of this ponderous proposition; but our apprehensions are again aroused when we read that "it is reserved for future generations of men to fill up this yawning chasm by the aid of a more thorough knowledge of nature, and to comprehend the sphere of the material world as a unit."

Next is propounded the conundrum, "What is the primary beginning of the organic?" To this query the following answer is made :

"An admirable English naturalist, Huxley, succeeded, in the year 1868, in making a marvelous discovery. The bottom of our seas, at the most considerable depths, is covered over, in large tracts, with a strange, slimy substance. When this thing, called the bathybius, is drawn up by the dredge and placed under the microscope—under that instrument which has conquered the mighty world of minuteness for natural science—a very peculiar image is presented to the astonished eye," etc., etc. This constitutes the first page. We shall inflict no further excerpts upon the "astonished eye" of the reader, but simply summarize our impressions of the book.

The style lacks simplicity, clearness, terseness—hence force; it is rich in ambiguity, elephantine vivacity and commencement-day rhetoric; complications of negatives and abrupt transitions abound; originality in the use of language is strikingly apparent. Talleyrand is credited with the remark that the art of conversation consists in concealing one's thoughts; our author would evidently not restrict the art to conversation.

Yet concealed beneath the grotesque manner is much valuable matter. The book is apparently a condensation of Frey's "Histology and Histo-Chemistry,"—which, though imperfect, is probably the best text-book on the subject in the language. The larger book is generally free from the follies of the smaller —thanks, probably, to the translator. The general plan of the "Compendium" is excellent, the admirable classification and arrangement of the larger work being substantially retained in the smaller. The first lecture is a brief discussion of the origin, structure and function of the cell; the second is devoted to classification of the tissues, based upon the variation of the component cells from their primitive type, and upon the nature of the intercellular substance. This classification the author clumsily, but truthfully, pronounces "preponderating artificial, which, with all its defectiveness, possesses, at least, the advantage of presenting the material in a more convenient form to the learner." Seven succeeding lectures are consumed in describing the individual tissues; the remainder of the work discusses the combination of those tissues in the formation of organs. It is to be regretted that but little attention is given to embryology; surely the structure of mature tissues and organs would be far more intelligible to the student, after a study of their origin and development.

The exposition in the compendium is, in general, sufficiently minute for the student, and yet so brief as to secure attention where the elaborate descriptions of Stricker and Frey would overwhelm.

For the fidelity of the work the author's name is sufficient guaranty; that he differs from other histologists on certain points is due to the fact that he has a foundation for opinions of his own.

The wood-engravings are more than ordinarily well executed. A singular omission, especially unfortunate to the student of theoretical history only, is the neglect to state the amplification represented in the cuts.

On the whole we do not regard the compendium as essential to the student's library. W. L. B.

THE ELECTRIC BATH—ITS MEDICAL USES, EFFECTS AND APPLIANCES. By *George M. Schweig, M.D.* 134 pages. New York: *G. P. Putnam's Sons.* 1877.

With the exception of a few meager remarks on the "electro-chemical bath," in one or two works on electricity, and a few fugitive articles in some of the recent journals, there has been but little recorded of the electrical bath as a remedial agent. Should we consult the patent office reports, and some of the proceedings of courts of justice, we would find that the electric bath is no new thing. This is but another illustration of the fact, that many of our best remedies, and now recognized means for the treatment of disease, have originated outside of the medical profession, and have only been adopted after their utility has been proven by the laity, and some physician has first used and given them to the profession.

This work from a scientific source, the first-born, though by no means the first conceived of the kind, we welcome, as an effective means of taking from the laity and charlatans a valuable, and frequently (in their hands) a dangerous means of treating disease.

The publishers have performed their work well, and have given us an attractive book.

The apparatus used and described by Dr. Schweig is crude, and admits of but few of the finer modes of application of the more complete apparatus. His tub, from the description, is but little more than a square box, for he says, "It is very difficult to make a wooden tub with a slanting back, water-tight." This is entirely different from our experience in the construction of tubs. He also says, "It must not be forgotten, however, that a wooden tub requires to be well painted inside, in order to prevent its becoming water-soaked, because in that event it would become a conductor of electricity, and interfere, to some extent, with the administration of the electric current." Here we again differ. Should the unpainted tub become water-soaked, it is not enough better conductor than water to make any perceptible difference in the passage of the current from one electrode to another through the water. Even if the tub is painted, it is liable to become water-soaked. We speak from experience, for we have used both the painted and unpainted tubs, and have found no difference. The electrodes, two in number, are fastened one at the head and the other at the foot of the tub, by means of a piece of cloth stretched over the electrode and tacked to the tub. This is certainly a most uncleanly means, for after a few days immersion in water, the cloth will become slimy and decidedly nasty, besides affording a splendid receptacle for that which the patient may leave behind him. The "surface board" (p. 14) could with advantage be dispensed with, and any of the ordinary forms of electrodes for the general local application of electricity be substituted with advantage.

He recognizes the great advantage derived from medicating the bath with various chemicals, the benefits of which can hardly be appreciated save by those who have had experience in this mode of administering remedies.

We regret that he does not enter more into the detail of treating the various cases he records. What he has said of the treatment will be of service to those who have no knowledge of the bath; yet we have the feeling as we finish the book, that because he knows all about the administration of the bath, his readers must also know all that he does, and thus supply that which is not written.

On page 97 occurs, "but will carefully discriminate, and conscientiously exclude those cases in which general electrization might result injuriously. In such cases a tolerably accurate diagnosis is, as a rule, readily made, and will enable the physician to separate the suitable from the unsuitable cases." That this is the fact we do not question, but we certainly would like some details of the cases in which injury may result, rather than the bare statement that such is the case.

We are glad this book has been added to medical literature, as it is, perhaps, the first of a series which will supply the needs of the profession.

